# A New Quaternion-Based Kalman Filter for Human Body Motion Tracking Using the Second Estimator of the Optimal Quaternion Algorithm and the Joint Angle Constraint Method with Inertial and Magnetic Sensors

**DOI:** 10.3390/s20216018

**Published:** 2020-10-23

**Authors:** Yingbo Duan, Xiaoyue Zhang, Zhibing Li

**Affiliations:** School of Instrumentation and Optoelectronic Engineering, Beihang University, Beijing 100191, China; duanyingbo@buaa.edu.cn (Y.D.); lizhibing@buaa.edu.cn (Z.L.)

**Keywords:** human body motion tracking, Kalman filter, the ESOQ-2 algorithm, quaternion, inertial/magnetic sensors

## Abstract

Human body motion tracking is a key technique in robotics, virtual reality and other human–computer interaction fields. This paper proposes a novel simple-structure Kalman filter to improve the accuracy of human body motion tracking, named the Second EStimator of the Optimal Quaternion Kalman Filter (E2QKF). The new algorithm is the combination of the Second Estimator of the Optimal Quaternion (ESOQ-2) algorithm, the linear Kalman filter and the joint angle constraint method. In the proposed filter, the ESOQ-2 algorithm is used to produce an observation quaternion by preprocessing accelerometer and magnetometer measurements. The compensation for the accelerometer added in the ESOQ-2 algorithm is to eliminate the influence of human body motion acceleration included in the results. The state vector of the filter is the quaternion, which is calculated with gyroscope measurements, and the Kalman filter is to calculate the optimal quaternion by fusing the state quaternion and the observation quaternion. Therefore, the filter becomes a simple first-order linear system model, which avoids the linearization error of measurement equations and reduces the computational complexity. Furthermore, the joint angle constraint is considered in the proposed algorithm, which makes the results more accurate. To verify the accuracy of the proposed algorithm, inertial/magnetic sensors are used to perform the upper limb motion experiment, and the result of E2QKF (without joint angle constraint) is compared with an optical motion capture system and two traditional methods. Test results demonstrate the effectiveness of the proposed filter: the root mean square error (RMSE) of E2QKF is less than 2.0° and the maximum error is less than 4.6°. The result of E2QKF (with joint angle constraint) is compared with E2QKF (without joint angle constraint). Test results demonstrate the superiority of E2QKF (with joint angle constraint): the joint angle constraint method can further improve the accuracy of human body motion tracking.

## 1. Introduction

As a key means of human–computer interaction, human body motion tracking is an important technique in virtual reality, robotics and other fields. According to the working principle, human body motion tracking can be separated into five categories: mechanical, optical, acoustic, electromagnetic and inertial/magnetic, and different measurement methods have their advantages and disadvantages [1].

Mechanical tracking systems consist of multiple joints and connecting rods, relying on mechanical devices to track human body motion. It has low cost, high precision and good real-time performance. The disadvantage is that its structure has a large restriction on human body motion, and the structure is cumbersome which is extremely inconvenient to use. Optical tracking systems can be separated into two categories: image-based systems and pattern recognition systems. Image-based systems consist of multiple markers and cameras, and they track human body motion by using cameras to track predesignated markers on moving objects within a working volume [2]. Pattern recognition systems use image recognition/analysis technology to let the visual system directly identify the main limb of the subject’s body and record their movements. In general, the advantages of the optical tracking system are small data transmission delay, good real-time performance and high measurement accuracy. However, the price of the optical tracking system is relatively expensive, the pre-calibration is cumbersome and post-data processing requires a lot of manual intervention. Acoustic tracking systems mainly consist of a sound wave transmitter and a sound wave receiver. Ultrasonic waves are transmitted from transmitter to receiver, so there is a time and phase difference. According to the difference, the motion trajectory of the measured object can be determined. This system, although low cost, has low accuracy and is susceptible to noise. Electromagnetic tracking systems are mainly composed of three parts: magnetic field emission source, magnetic receiving sensor and data processing unit. The system has good real-time performance and low cost. However, its shortcomings are also obvious. Since the magnetic field is easily disturbed, it requires no heavy metals in the measurement environment, otherwise the accuracy will be affected. In addition, the cable creates many restrictions on human body motion.

With the rapid development of Micro-Electro-Mechanical System (MEMS) sense technology, a large number of inertial measurement units with low cost and high precision appeared in the market, which provide an opportunity for inertial/magnetic tracking technology to improve accuracy and reduce cost. As a kind of human body motion tracking technology with great development potential, they have received wide attention in recent years. Inertial/magnetic tracking systems refer to the use of gyroscopes, accelerometers, magnetometers and other small MEMS units to track human body motion through wireless sensor networks, which are convenient to use and have low requirements for the environment. Based on an inertial sensor, [3] proposed a human attitude determination algorithm, aided by compensation filtering. Multiple lidars and inertial sensors were used to track the real-time pose of human motion [4].

There are several points of inertial/magnetic tracking technology that should be considered: initial calibration, data fusion, interference of human body motion acceleration, interference of magnetic field, the sensors mounting position, etc. The most critical one is the sensor data fusion problems [5,6]. In the MEMS sensor, which constitutes the inertial/magnetic tracking system, the rate gyros can be used to estimate human body motion separately [7,8]. With this method, however, the error will accumulate over time. The combination of an accelerometer and a magnetometer can also measure human body motion [9]. However, the accelerometer is not only sensitive to gravitational but also human body motion acceleration, and the magnetometer is susceptible to ferromagnetic substances and external magnetic fields so that the combination can only be used in the case of slow human body motion and a stable magnetic field. The authors of [10] proposed a gyroscope-based algorithm with accelerometer correction to track the desired body portion without considering the influence of the magnetic field. In summary, the combination of the above three sensors is typically used in inertial/magnetic tracking systems. 

There are three commonly used methods for the fusion of data from the incorporated sensors: Kalman filter, complementary filter and the gradient descent algorithm. Kalman filtering mainly uses data from accelerometers and magnetometers to compensate for attitude errors caused by the gyroscope. It has relatively high-precision calculation results but requires higher hardware performance due to its computational complexity [11]. The complementary filter eliminates the low-frequency noise generated by the zero offset of the gyroscope and other errors, and filters out the high-frequency noise contained in the accelerometer and magnetometer, and complements the above two calculated attitude information to obtain the final accurate attitude [12]. Its structure is simple and easy to design, but the difficulty is that its conversion frequency is difficult to determine accurately. The gradient descent algorithm describes the error of attitude measurement between the gyroscope, accelerometer and magnetometer through the constructor f(x). The attitude estimation is obtained by successive generations in the anti-gradient direction [13]. The calculation accuracy may be reduced under intense motion.

This paper proposed a simple structure Kalman filter for inertial/magnetic high-precision human body motion tracking. In order to obtain stable and comprehensive human body motion data, nine-axis inertial/magnetic sensors containing a tri-axis gyroscope, accelerometers and magnetometers were used in this paper. Quaternions are used to represent human body motion rather than Euler angles. The purpose of using quaternions is that they do not suffer from the singularity problem and can avoid trigonometric functions, making them more computationally efficient and easier to implement in real time on microcontrollers. After the optimal quaternion was calculated, it was transformed into Euler angles for a visual representation. The ESOQ-2 algorithm is used to preprocess accelerometer and magnetometer measurements, resulting in a significant simplification of the Kalman filter design. Furthermore, the compensation of the accelerometer is added in the ESOQ-2 algorithm to eliminate the influence of human body motion acceleration on the results. The ESOQ-2 is a very effective fast attitude estimation algorithm [14] and can provide an accurate reference quaternion as the observation vector of the Kalman filter. The state vector of the Kalman filter is the quaternion which is calculated with gyroscope measurements. It can be seen from above that the Kalman filter becomes a first-order linear system. Then, the Kalman filter was used to fuse the state quaternion and the observation quaternion to calculate the optimal quaternion of the human body attitude. In addition, we considered the human body joint angle constraint during human body motion tracking, which makes the results more accurate. In the end, the filter’s performance is validated by experimental results, which demonstrate that it is suitable for high-precision human body motion tracking.

The structure of the paper is as follows. In Section 2, the basic definitions and details of the proposed algorithm will be described. The upper limb motion measurement experiment results are presented and discussed in Section 3. Section 4 is the Conclusions.

## 2. Materials and Methods

### 2.1. Coordinate System Definition

**Figure 1:**
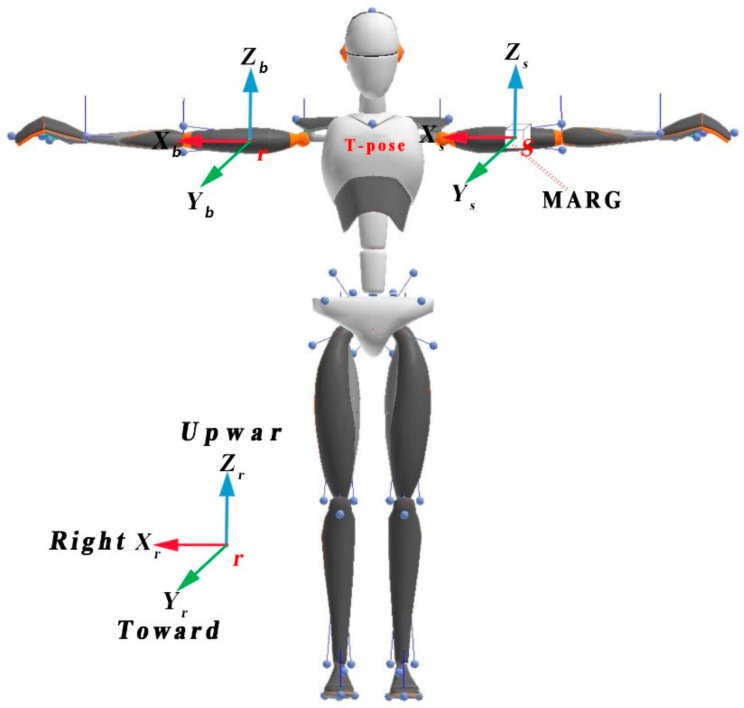
The definitions of the coordinate frames.

This paper conducts related research on inertial/magnetic human body motion tracking. Inertial/magnetic navigation is based on the precise definition of a series of Cartesian reference coordinate systems [15], and the motion of the human body in space is obtained by the conversion relationship between different coordinate frames. Therefore, to represent the human body motion attitude, the following definition and description of the relevant coordinate frames in this paper are required. The definition of the coordinate frames involved in this paper is illustrated in Figure 1. The reference coordinate frame mounted on the earth was defined as r, the limb coordinate frame was defined as b and the magnetic, angular rate and gravity (MARG) sensor coordinate frame was defined as s.

The calibration procedures for the accelerometer, gyroscope and magnetometer are carried out according to [16].

- The reference coordinate frame *r*

The description of the human body motion attitude in this paper is relative to the reference coordinate system. Once the reference coordinate frame is established, it will not change with human body motion. The z-axis of the reference coordinate frame points to the sky along the direction of the gravity vector, the x-axis points to the right of the human body, the y-axis points to the front of the human body and the three axes follow the principle of the right-handed rectangular coordinate frame.

- The limb coordinate frame *b*

According to different limbs of the human body, the limb coordinate frame can be subdivided into shoulder limb coordinate frame, arm limb coordinate frame, thigh limb coordinate frame, etc. The origin of the limb coordinate frame is selected at the geometric center of the limb, the x-axis points to the right of the human body, the y-axis points to the front of the human body, the z-axis points to the sky along the direction of the gravity vector and the three axes follow the principle of the right-handed rectangular coordinate frame. The limb coordinate system and the reference coordinate system maintain the same direction of each axis during the initial posture calibration stage of the human body, and there is a translation transformation relationship between them in space.

- The MARG sensor coordinate frame *s*

The origin of the MEMS sensor coordinate system is selected as the center of gravity of the sensor. The axis is parallel to the two right-angle sides of the sensor plane and perpendicular to each other. The axis is perpendicular to the plane where the axis lies and points to the sky.

For the sake of simplicity, we assumed that there was no relative displacement between the sensor and limb during the motion of the human body, so the sensor coordinate frame s and limb coordinate frame b were considered to be identical. 

### 2.2. Representation

Quaternions are used to represent the orientation of each body limb segment in the proposed filter. The advantage of using quaternions is that they do not suffer from the singularity problem and can avoid trigonometric functions, making them more computationally efficient and easier to implement in real time on microcontrollers. The general form of the quaternion expression is [17]

(1) $$\dot{q}=\frac{1}{2}q⊗\omega$$

where $\dot{q}$ is the quaternion derivative, ⊗ represents quaternion multiplication,$\;\omega ={\left[0\;\;\omega_{b}\right]}^{T}$ is a four-element column vector and $\;\omega_{B}={\left[\omega_{Bx\;}\omega_{By}\;\omega_{Bz}\right]}^{T}$ is the angular rates for the x-, y- and z-axis in the limb coordinate frame b.

**Figure 2:**
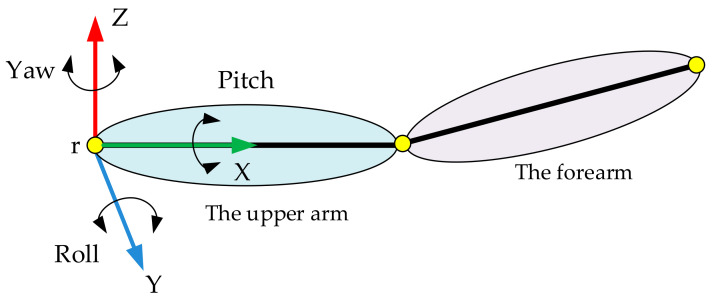
The definition of Euler angles.

The definition of Euler angles of human body motion in this paper is shown in Figure 2. For example, we chose the upper limb as the research object. The yaw angle is a rotation angle with respect to the z-axis; the roll angle is a rotation angle with respect to the y-axis; and the pitch angle is a rotation angle with respect to the x-axis.

### 2.3. Data Fusion Based on a Kalman Filter

**Figure 3:**
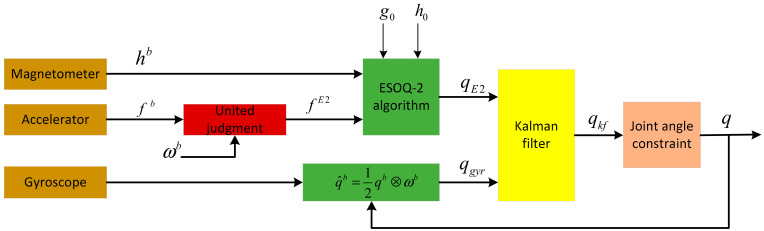
Block diagram of the proposed filter.

We proposed a Kalman filter based on the ESOQ-2 algorithm. Figure 3 shows the block diagram of the Kalman filter. It can be seen that the measurements of the accelerometer and magnetometer were used as the input vectors for the ESOQ-2 algorithm to produce the observation quaternion. The observation quaternion has a more precise dynamic response at low frequency, so it was used as the observation state for the Kalman filter to correct the predicted state obtained with the gyroscope. Finally, the computed quaternion by the filter was corrected with the joint angle constraint method to the results more accurately.

#### 2.3.1. The ESOQ-2 Algorithm

In 1965, Wahba proposed the Wahba problem, he estimated the attitudes of a spacecraft based on the vector’s reference information. The ESOQ-2 algorithm has been proposed for the Wahba problem. The core of the problem is to solve the optimal orthogonal matrix with determinant +1, making the minimum loss function as follows [18].

(2) $$L\left(A\right)=\frac{1}{2}\overset{n}{\underset{i-1}{\sum }}\alpha_{i}||b_{i}-A\gamma_{i}||{}^{2}=min$$

where $\alpha_{i}\;\left(\sum_{i}\alpha_{i}=1,i=1\sim 2\right)$ is the relative weight of the observation vector and it was determined by the data credibility of the observation vector; b is the vector defined in the body coordinate; γ is the vector defined in the reference coordinate frame; and A is the attitude matrix from γ to b. In this paper, b is the accelerometers and magnetometers data. 

In 1968, Davenport proposed the q-method, causing the Wahba problem to achieve a breakthrough. The core of the q-method is using the quaternion parameterized attitude matrix to transform the Wahba problem to the maximum eigenvalue problem of the matrix K [19].

(3) $$Kq=\lambda_{max}q$$

Therefore, the core of the problem is how to obtain the optimal attitude quaternion. The steps of solving the optimal attitude quaternion q are as follows:

a. The first step: calculate the matrix K:

(4) $$K=\left[\begin{array}{cc} V+V^{T}-tr\left[V\right]I_{3\times 3} & z\\ z^{T} & tr\left[V\right] \end{array}\right]$$

where V represents the attitude profile matrix and $V=\sum_{i=1}^{n}\alpha_{i}b_{i}r_{i}{}^{T}$ and $z=\sum_{i=1}^{n}\alpha_{i}b_{i}\times \gamma_{i}$.

b. The second step: calculate the maximum eigenvalue of the matrix K:

(5) $$\lambda =\frac{1}{2}\left(\sqrt{2\sqrt{d}-b}+\sqrt{-2\sqrt{c}-b}\right)$$

where $b=-2\left(tr\left[V\right]\right)+tr\left[adj\left(V+V^{T}\right)\right]-z^{T}z$ and $c=\det\left(K\right).$

c. The third step: calculate the optimal attitude quaternion q:

(6) $$q=\left\{\begin{array}{c} \left(\lambda_{max}-tr\left[V\right]\right)e_{k}\\ z^{T}e_{k} \end{array}\right\}$$

where $e_{k}$ represents the best robustness vector.

**Figure 4:**
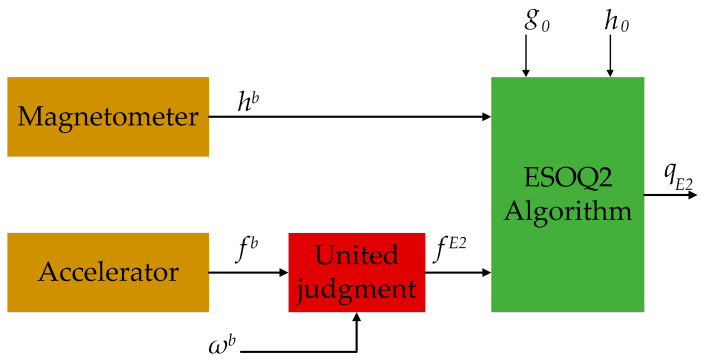
Block diagram of the ESOQ-2 algorithm.

The implementation of the ESOQ-2 algorithm is depicted in Figure 4. Firstly, the measured vector of the gravity field and magnetic field in slow motion conditions is given by

(7) $$\left\{\begin{array}{c} f^{b}=\frac{g^{b}}{\left\|g^{b}\right\|}\\ h^{b}=\frac{m^{b}}{\left\|m^{b}\right\|} \end{array}\right.$$

where $g^{b}=\left[a_{x}\;a_{y}\;a_{z}\right]$ and $m^{b}=\left[m_{x}\;m_{y}\;m_{z}\right]$ represent the acceleration and magnetic measurements in the limb coordinate frame b, respectively.

Then, we considered that the prerequisite for using the ESOQ-2 algorithm is that the environment should be quasi-static. However, the accelerometer is not only sensitive to gravity acceleration but also the motion acceleration in human body motion. When the motion acceleration is low, the accuracy of the ESOQ-2 algorithm is still credible. However, when the motion is highly dynamic, the accuracy of the ESOQ-2 algorithm drops, and the state quaternion is more reliable. In this case, we judged the motion acceleration. When the motion acceleration is low, the gravity acceleration vector input to the ESOQ-2 algorithm is the outputs of the accelerometer. When the motion acceleration is high, the input was replaced by the vector calculated with the input quaternion.

Therefore, the gravity acceleration vector input $f^{E2}$ to the ESOQ-2 algorithm was calculated using the following equations:

(8) $$\;f^{E2}=\left\{\begin{array}{c} T_{r}^{b}\frac{g_{0}}{\left\|g_{0}\right\|}:\left|\|g^{b}\|{-\|g}_{0}\|\right|>\delta_{a}\;or\;\|\omega_{b}\|>\delta_{\omega }\;\\ \frac{g^{b}}{\left\|g^{b}\right\|}:else \end{array}\right.$$

where $g_{0}={\left[\begin{array}{ccc} 0 & 0 & 9.78 \end{array}\right]}^{T}$ is the gravity vector; $T_{r}^{b}$ represents the transformation matrix from the reference coordinate frame r to the limb coordinate frame b**;** and $\delta_{\omega }=10^{{}^{\circ}}/s$ and $\delta_{a}=0.1m/s^{2}$ are the corresponding thresholds for acceleration and angular velocity, respectively.

#### 2.3.2. Process Model

We chose the quaternion which calculated with the gyroscope measurements as the state vector of the Kalman filter. The state vector is 4D, and the four components can be expressed as

(9) $$\left[\begin{array}{c} x_{1}\\ x_{2}\\ x_{3}\\ x_{4} \end{array}\right]=\left[\begin{array}{c} q_{0}\\ q_{1}\\ q_{2}\\ q_{3} \end{array}\right]=q_{gyr}$$

where $x_{i}\left(i=1,2,3,4\right)$ is the state quantity of the Kalman system.

Assuming that the measurement of the gyroscope is $\omega =\bar{\omega }+\delta \omega$, $\bar{\omega }={\left[{\bar{\omega }}_{x\;}\;{\bar{\omega }}_{y}\;{\bar{\omega }}_{z}\right]}^{\;T}$ is the ideal value and $\delta \omega ={\left[\delta \omega_{x\;}\;\delta \omega_{y}\;\;\delta \omega_{z}\right]}^{\;T}$ represents the drift of the gyroscope, then the state equation can be written as follows:

(10) $$\left[\begin{array}{c} \begin{array}{c} {\dot{x}}_{1}\\ {\dot{x}}_{2} \end{array}\\ \begin{array}{c} {\dot{x}}_{3}\\ {\dot{x}}_{4} \end{array} \end{array}\right]=\frac{1}{2}\left[\begin{array}{cccc} 0 & -{\bar{w}}_{x} & -{\bar{w}}_{y} & -{\bar{w}}_{z}\\ {\bar{w}}_{x} & 0 & {\bar{w}}_{z} & -{\bar{w}}_{y}\\ \omega_{y} & -\;{\bar{w}}_{z} & 0 & {\bar{w}}_{x\;}\\ {\bar{w}}_{z} & {\bar{w}}_{y} & -{\bar{w}}_{x\;} & 0 \end{array}\right]\left[\begin{array}{c} q_{0}\\ q_{1}\\ q_{2}\\ q_{3} \end{array}\right]+\frac{1}{2}\left[\begin{array}{ccc} q_{1} & q_{2} & q_{3}\\ -q_{0} & q_{3} & -q_{2}\\ -q_{3} & q_{0} & q_{1}\\ q_{2} & -q_{1} & -q_{0} \end{array}\right]\left[\begin{array}{c} \delta \omega_{x}\\ \delta \omega_{y}\\ \delta \omega_{z} \end{array}\right]$$

The next step is to convert the continuous-time model into the discrete-time model, and it can be described as

(11) $$x_{k+1}=\Phi_{k}x_{k}+w_{k},\;k=0,1,2,3,...$$

where $\Phi_{k}$ is the discrete-state transition matrix and can be described as formula (12);$\;x_{k}$ and $x_{k+1}$ are the quaternions at time kΔt and $\left(k+1\right)\Delta t$, respectively; and $w_{k}$ is the process noise, where the covariance matrix of it can be obtained by formula (13).

(12) $$\Phi_{k}=\left[\begin{array}{cccc} 1 & \frac{\Delta t}{2}\omega_{x} & \frac{\Delta t}{2}\omega_{y} & \frac{\Delta t}{2}\omega_{z}\\ \frac{\Delta t}{2}\omega_{x} & 1 & \frac{\Delta t}{2}\omega_{z} & \frac{\Delta t}{2}\omega_{y}\\ \frac{\Delta t}{2}\omega_{y} & \frac{\Delta t}{2}\omega_{z} & 1 & \frac{\Delta t}{2}\omega_{x}\\ \frac{\Delta t}{2}\omega_{z} & \frac{\Delta t}{2}\omega_{y} & \frac{\Delta t}{2}\omega_{x} & 1 \end{array}\right]$$

(13) $$\;\omega_{k}=\frac{\Delta t}{2}\left[\begin{array}{c} \begin{array}{ccc} \;\;\;q_{1} & \;q_{2} & \;\;q_{3}\\ \;-q_{0} & \;\;\;q_{3} & \;-q_{2} \end{array}\\ \begin{array}{ccc} -q_{3} & -q_{0} & \;\;\;\;q_{1}\\ \;\;\;\;q_{2} & \;-q_{1} & \;-q_{0} \end{array} \end{array}\right]v_{gk}$$

where Δt represents the sample time (we set it to 0.01 s in our implementation); and $v_{gk}$ is the mutually uncorrelated zero-mean white Gaussian noise with covariance matrix $\sum_{g}\delta_{3\times 3}^{2}$. Then, the process noise covariance matrix $Q_{k}$ is presented as follows:

(14) $$Q_{k}=E\left(\;\omega_{k}\;\omega_{k}{}^{T}\right)$$

where E is the expectation operator; then, the experimentally determined values are

(15) $$Q_{k}=\mathrm{diag}\left(1\times 10^{-6},1\times 10^{-6},1\times 10^{-6},1\times 10^{-6}\right)$$

#### 2.3.3. Observation Model 

The acceleration and magnetic measurements were used as the input vectors for the ESOQ-2 algorithm to produce the observation quaternion:

(16) $$\left[\begin{array}{c} z_{1}\\ z_{2}\\ z_{3}\\ z_{4} \end{array}\right]=\left[\begin{array}{c} eq_{0}\\ eq_{1}\\ eq_{2}\\ eq_{3} \end{array}\right]=q_{ref}$$

where $eq_{i}\left(i=1,2,3,4\right)$ is the quaternion solved by ESOQ-2.

The measurement equation is given by

(17) $$z_{k}=H_{k}x_{k}+v_{k}$$

where $H_{k}$ is the 4 × 4 identity matrix; and $v_{k}$ is the measurement noise, and its covariance matrix is given by

(18) $$R_{k}=E\left(\;v_{k}\;v_{k}{}^{T}\right)$$

The variances of the reference quaternion components are determined using computed quaternions. The experimentally determined values are

(19) $$R_{k}=diag\left(0.0001,0.0001,0.0001,0.0001\right)$$

#### 2.3.4. Kalman Filter Fusion

After the discrete process (11) and the discrete measurement (17) are obtained, we still need to determine the initial estimate quaternion ${\hat{x}}_{0}$ and the initial covariance matrix $P_{0}$ to design the KF. The initial estimate quaternion ${\hat{x}}_{0}$ can be calculated using the initial data by the adaptive ESOQ-2 algorithm. The initial covariance matrix $P_{0}$ is determined so that $P_{0}=\mathrm{diag}\left(0.01,0.01,0.01,0.01\right)$.

Then, according to the Kalman filter theory, the optimal state estimation of the system can be obtained by the iteration of time and measurements update. The Kalman filter recursive process is as follows [20]:

a. Project the state and covariance estimates from time step k−1 to step k:

(20) $$\;\left\{\begin{array}{c} {\hat{X}}_{k+1}^{-}=\Phi_{k}X_{k}\\ P_{k+1}^{-}=\Phi_{k}P_{k}\Phi_{k}{}^{T}+Q_{k} \end{array}\right.$$

b. Calculate the Kalman gain:

(21) $$\;\;K_{k+1}=P_{k+1}^{-}{\left(P_{k+1}^{-}+R_{k+1}\right)}^{-1}$$

c. Calculate the posterior error covariance estimate:

(22) $$\;\;\;\left\{\begin{array}{c} {\hat{X}}_{k+1}={\hat{X}}_{k+1}^{-}+K_{k+1}\left(Z_{k+1}-{\hat{X}}_{k+1}^{-}\right)\\ P_{k+1}=(I-K_{k+1})P_{k+1}^{-} \end{array}\right.$$

where $Z_{k+1}$ is the computed quaternion given by Equation (17).

From the process of the Kalman filter mentioned above, we can calculate the optimally estimated quaternion and obtain the attitude of human body motion.

### 2.4. The Human Body Joint Angle Constraint Method

Human physiological characteristics need to be considered in human body motion tracking. Due to the measurement error in the motion attitude of a single limb, when the motion attitude of the human body is considered to be combined only by the attitude of each single limb, it will make the final motion attitude of the human body lose its authenticity. Therefore, it is necessary to study the measurement method of the human body motion attitude in combination with the human physiological structure. It can be seen from the human physiological characteristics that the human body limbs are connected through the joints of the endpoints of its bones, and the most obvious physiological characteristics of the human body are the joint angle between the individual limbs. Therefore, the joint angle is used as the physiological characteristics of the human body to further study the human body motion tracking method.

The upper limb has the greatest freedom of movement in each limb [21,22], so the upper limb is selected as the object to carry out the relevant research.

#### 2.4.1. Introduction to Human Sports Anatomy Terminology

**Figure 5:**
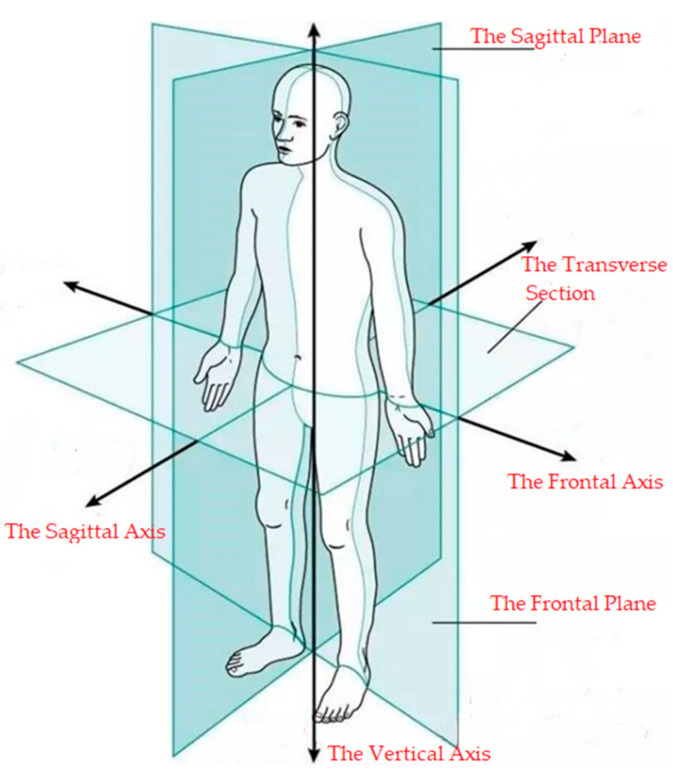
Fundamentals and basic axes of the human body.

Before studying the physical characteristics of the human upper limb, we need to introduce the concepts and terms in human body motion anatomy. As shown in Figure 5, based on the human anatomy, the human body has three mutually perpendicular basic planes and axes, which can define the movement of the human limbs and joints [23]. The sagittal plane is perpendicular to the ground and can divide the human body into two symmetrical parts in the left-to-right direction. The section along the left–right direction of the body and perpendicular to the ground and dividing the human body into two parts is called the frontal plane. The section along the vertical direction of the body and parallel to the ground which divides the human body into upper and lower parts is called the transverse section. The axis along the front–back direction of the body and perpendicular to the frontal plane is called the sagittal axis. The axis that passes vertically through the sagittal plane is called the frontal axis. The axis that passes vertically through the cross-section of the body is called the vertical axis.

In the human motion anatomy, the limb movements are mainly divided into three categories according to the movement of the joints: flexion/extension movement refers to the movement of the limb around the frontal axis in the sagittal plane; adduction/abduction movement refers to the movement of the limb around the sagittal axis in the frontal plane; inside-out/outside-out means that the limb rotates about its vertical axis in the cross-section. 

#### 2.4.2. Analysis of Physiological Characteristics of the Upper Limb

The upper limb is a multi-degree-of-freedom motion system composed of many complex-structure joints. It mainly includes three joints: shoulder joint, elbow joint and wrist joint, and three single limbs: upper arms, forearm and hand. The shoulder joint is the most flexible and most complex joint among all joints. It can perform various movements such as flexion/extension, adduction/abduction and internal rotation/external rotation, and it has three degrees of freedom in rotation. The elbow joint is a synovial hinge joint. The wrist joint is generally considered to have two degrees of freedom of flexion/extension and adduction/abduction. The movement of each limb in the upper limb is formed by stretching and contracting the muscles to move their corresponding bones around the joint. In this paper, the upper limb model is simplified, so the elbow joints and the lower arm are selected for related research.

Due to the physiological characteristics of the human body, the movement angle of each joint in the human body has a corresponding limit. Table 1 lists the range of motion angles of the joints in the upper limb. It should be noted that the data given in Table 1 are for normal adult males. The data will be different according to the age and gender of the person.

#### 2.4.3. The Joint Angle Constraint Method

It is not only the attitude of each limb that needs to be measured but also the angle of each joint in the human body needs to be obtained in human body motion tracking. Therefore, the elbow joint is used as an example to study the method of calculating the joint angle in this paper. According to the international biomechanics’ standard [25], each joint angle of the human body is defined according to the movement of the lower limb of the joint relative to the adjacent upper limb. Therefore, the angle of the elbow joint can be regarded as the forearm relative to the upper arm.

Using the E2QKF algorithm to calculate the quaternion and of the attitude of the forearm and upper arm, the elbow joint angle rotation quaternion is [26]

(23) $$q_{joint}={\left(q_{r}^{b1}\right)}^{-1}⊗q_{r}^{b2}$$

After the elbow joint angle rotation quaternion was calculated, the corresponding Euler angles of the elbow joint could be obtained. The pitch angle θ in Euler angles represents the angle of internal rotation and external rotation of the joint, the roll angle γ represents the angle of adduction and abduction of the joint and the heading angle φ represents the angle of flexion and extension of the joint.

There is an unavoidable measurement error in the single-limb motion posture measurement method E2QKF, and this error may cause the final human body motion attitude to lose its authenticity. Therefore, we considered the joint angle constraint, which makes the results more in line with the actual human physiological structure.

It can be seen from Table 1 that the elbow joint has two degrees of freedom in rotation, namely flexion/extension (130**°**/0**°**) and internal rotation/external rotation (−85**°**/85**°**). Therefore, in an ideal case, the Euler angles obtained from the quaternion of the elbow joint angle should have a pitch angle of −85°~85°, a roll angle of 0° and a heading angle of 0°~130°.

According to the above analysis, the elbow joint Euler angles have corresponding limits, and the boundary values can be constrained. The constraint method is as follows: First, the elbow joint angle is solved according to the quaternion of the upper arm and forearm attitude quaternion and converted to Euler angles. Then, we can judge whether the values are following the physiological characteristics of the human body. If they are within the corresponding value range, the normal solution result is maintained; otherwise, the Euler angles which are outside the value range are set to the closest angle of its boundary value. For example, if the heading angle φ in the elbow joint is calculated to be 132° during the measurement process, the heading angle is immediately taken to be 130° and denoted as $\varphi_{0}$, and the quaternion of the elbow joint angle $q_{joint0}$ corresponding to the heading angle $\varphi_{0}$ can be calculated using the following equation:

(24) $$q_{joint0}={\left[\begin{array}{c} \begin{array}{c} cos\left(\frac{\varphi_{0}}{2}\right)cos\left(\frac{\theta }{2}\right)cos\left(\frac{\gamma }{2}\right)-sin\left(\frac{\varphi_{0}}{2}\right)sin\left(\frac{\theta }{2}\right)sin\left(\frac{\gamma }{2}\right)\\ cos\left(\frac{\varphi_{0}}{2}\right)sin\left(\frac{\theta }{2}\right)cos\left(\frac{\gamma }{2}\right)-sin\left(\frac{\varphi_{0}}{2}\right)cos\left(\frac{\theta }{2}\right)sin\left(\frac{\gamma }{2}\right) \end{array}\\ \begin{array}{c} cos\left(\frac{\varphi_{0}}{2}\right)cos\left(\frac{\theta }{2}\right)sin\left(\frac{\gamma }{2}\right)+sin\left(\frac{\varphi_{0}}{2}\right)sin\left(\frac{\theta }{2}\right)cos\left(\frac{\gamma }{2}\right)\\ cos\left(\frac{\varphi_{0}}{2}\right)sin\left(\frac{\theta }{2}\right)sin\left(\frac{\gamma }{2}\right)+sin\left(\frac{\varphi_{0}}{2}\right)cos\left(\frac{\theta }{2}\right)cos\left(\frac{\gamma }{2}\right) \end{array} \end{array}\right]}^{T}$$

Then, the steps of the joint angle constraint method are as follows:

- Use the proposed E2QKF algorithm to calculate the quaternion of the upper arm and the forearm and calculate the quaternion of the elbow joint angle according to Equation (23).
- Convert the elbow joint angle quaternion to Euler angles, and judge whether the Euler angles value at each moment conforms to the physiological characteristics of the elbow joint.
- If the Euler angles value meets the physiological characteristics of the elbow joint, no processing will be performed. Otherwise, the Euler angles value at the time (set as time t) is set as the boundary value, and the corrected forearm attitude quaternion $p_{r}^{b2}$ can be calculated according to formula (23) and the quaternion of the elbow joint boundary angle, as shown in the following formula:

(25) $$p_{r}^{b2}=q_{r}^{b1}⊗q_{joint0}$$

d. Let $p_{r}^{b2}$ be the forearm attitude quaternion at time t and participate in the subsequent attitude estimation as the state estimation in E2QKF. Besides, since the system sample time is very small, the joint angle quaternion at time t + 1 can be set as $q_{joint0}$ then the corrected upper arm attitude quaternion at time t + 1 is

(26) $$q_{1r}^{b1}=q_{1r}^{b2}⊗q_{joint0}^{-1}$$

Through the above steps, the attitude correction of the upper arm, the forearm and the elbow joint is completed.

## 3. Experiment

To verify the proposed algorithm, an upper limb motion experiment was carried out. We evaluated the performance of the algorithm at different test motion speeds and times, and we also compared the algorithm with Yun’s method [2] and the method of [27] which was proposed by the author of this paper to further evaluate its performance.

### 3.1. Experimental Setup

In human body motion, the upper limb is more flexible and agile than the other body limbs [21]. In past related research, many researchers selected the upper limb as the main research object [2,21,22,27].

**Figure 6:**
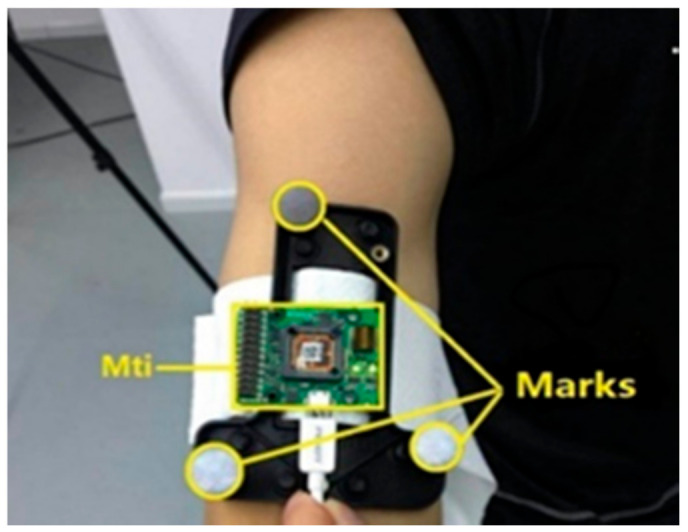
The MTi-3 and optical marks.

**Figure 7:**
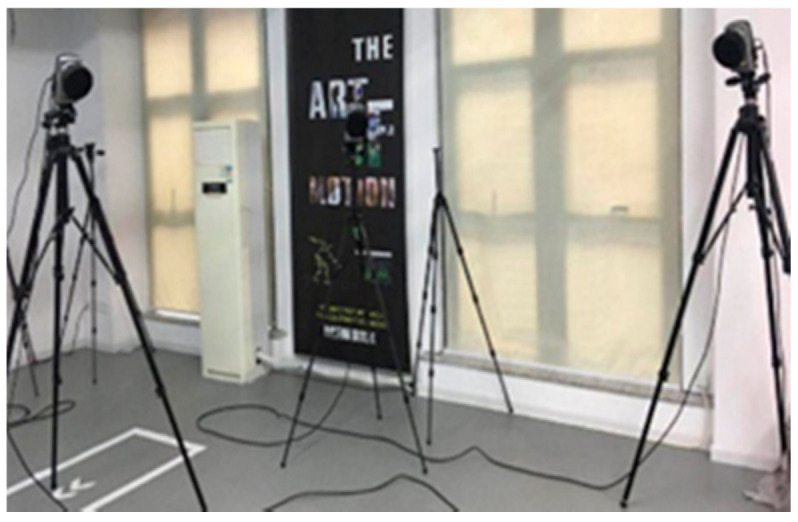
Oqus 6+.

**Figure 8:**
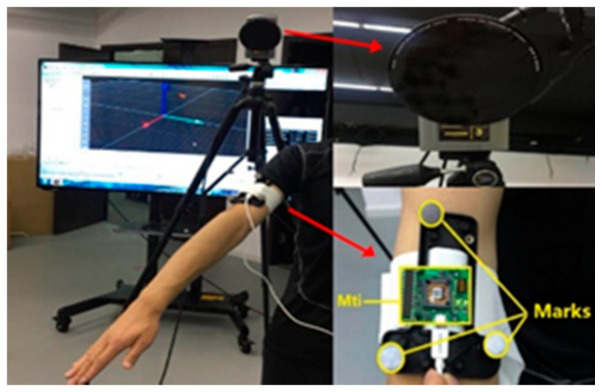
The right upper limb experiment.

A high-precision sensor, Mti-3 [28], was used in the experiment, which includes a tri-axis gyroscope, a tri-axis accelerometer and a tri-axis magnetometer. Figure 6 shows the subject with the MTi-3 bound to his upper arm, and three circular white mark points were fixed around the sensor for optical motion measurement. The optical measurement system Oqus6+ [29] is shown in Figure 7, which uses three cameras to measure the upper limb motion by using passive reflective technology and provides a standard reference orientation. The spatial positioning accuracy of Oqus 6+ is less than 1 mm, and the latency time is less than 4 ms. The right upper limb experiment is shown in Figure 8.

**Figure 9:**
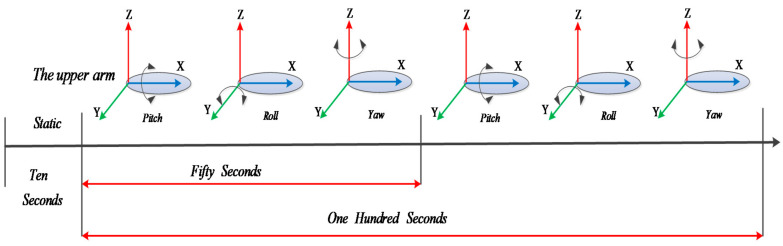
The procedure of limb motion.

In human body motion tracking, we found that the indicators for evaluating the performance of the proposed filter are divided into two aspects: The first is whether the method can adapt to different test times, in other words, whether it can adapt to long-term measurements. The other is whether the method can adapt to different motion speeds. Based on the above considerations, we performed experiments at different times and speeds to test the performance of the proposed filter. In the experiment, the subject swung his upper arm according to the procedure of Figure 9.

As Figure 9 shows, the subject remained static under T-pose for ten seconds to calculate the initial position. Then, he swung his upper arm in the order of x–y–z-axis of the reference coordinate system (pitch–roll–yaw) for about 55 s and subsequently repeated the motion in the same order. At last, the subject stretched his upper arms and remained static under T-pose for about 10 s. The whole experiment is called the 100-s test, and the first part of 100-s test is called the 50-s test. By comparing the results of 50-s test and 100-s test, we can evaluate the effect of test time on the proposed algorithm.

The maximum movement frequency of the upper arm is about 3.7 times per second [30]. To evaluate the effect of different speeds on the proposed filter, we chose two speeds in this study: 0.5 movements per second (low-speed) and 2 movements per second (high-speed). All of the above experiments were performed three times to ensure the credibility of the results. 

**Figure 10:**
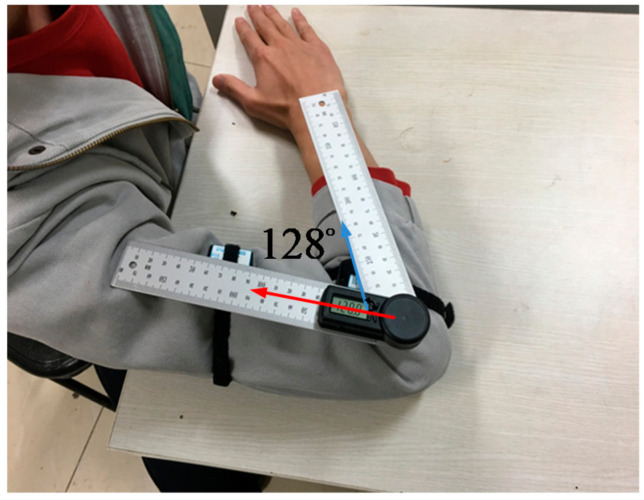
The relative posture of the upper arm and the forearm.

In practice, the flexion/extension motion of the elbow joint is relatively easy to complete when compared to the internal rotation/external rotation motion and it has a high frequency in daily life. Further, the trigger condition of the constraint method is that the calculated elbow joint angles exceed the boundary. Therefore, to test the effect of the joint angle constraint method, the forearm is moved following the upper arm and keeping the elbow joint to maintain the maximum flexion movement angle. As shown in Figure 10, the upper arm and the forearm always move at the relative angle in the experiment, and the maximum angle of the elbow joint flexion movement of the person was 128°.

In this study, we chose the root mean square error (RMSE) value of the Euler angles to verify the estimation accuracy of the filter [2,21,22,27]. The calculation formula is as follows:

(27) $$\mathrm{RMSE}=\sqrt{\frac{\sum_{i=1}^{n}{\left(\beta_{test}-\beta_{optic}\right)}^{2}}{n}}$$

where $\beta_{test}$ represents the Euler angles calculated by the filter; $\beta_{optic}$ represents the Euler angles measured by the Oqus 6+; and n represents the number of samples.

### 3.2. Results and Discussion

#### 3.2.1. Movement Test

**Figure 11:**
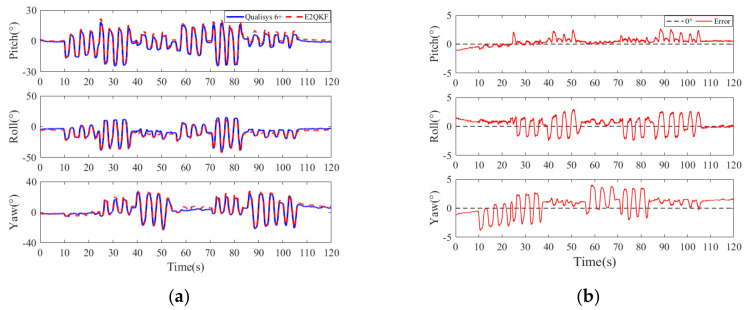
Typical E2QKF (without joint angle constraint) measurement results of the upper arm at low speeds. (**a**) The Euler angles of the Oqus 6+ and E2QKF (without joint angle constraint). (**b**) Euler angle errors.

**Figure 12:**
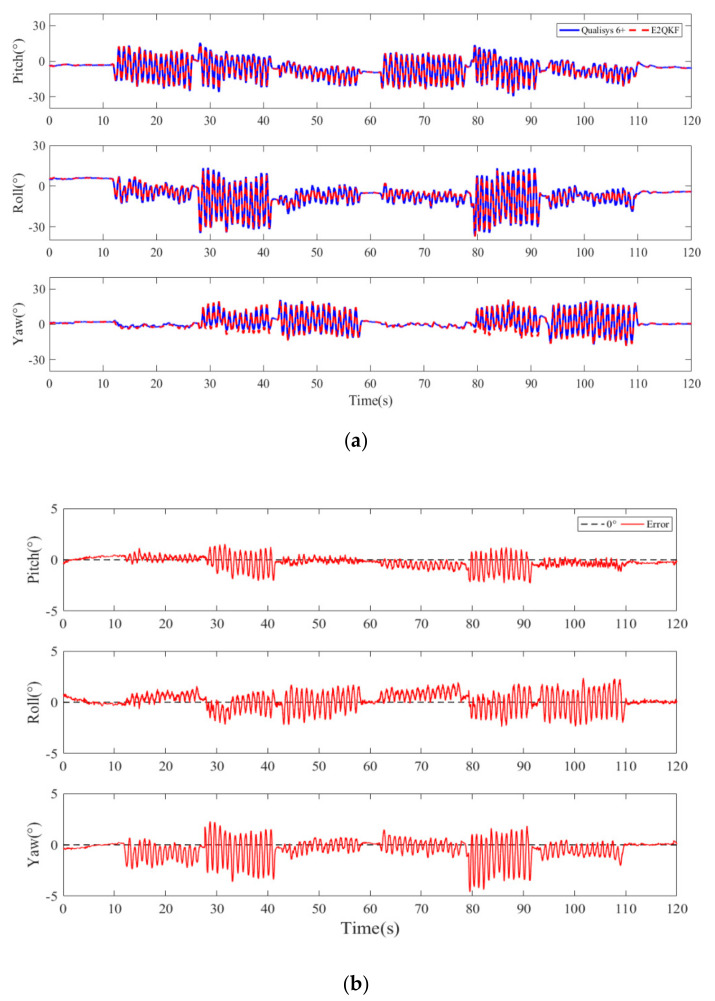
Typical E2QKF (without joint angle constraint) measurement results of the upper arm at high speeds. (**a**) The Euler angles of the Oqus 6+ and E2QKF. (**b**) Euler angle errors.

Figure 11a,b are typical E2QKF (without joint angle constraint) measurements of the upper arm at 0.5 movements per second (low speeds). Figure 12a,b are typical measurements of the E2QKF (without joint angle constraint) measurements of the upper arm at 2 movements per second (high speeds). The two experiments are shown as the measurement time of 120 s, in which 0 to 10 s is the initial attitude calibration time; 10 to 60 s and 60 to 110 s are repeat movements, and 110 to 120 s is the static time. Especially, in Figure 11a and Figure 12a, the blue solid lines represent the Euler angles measured by the Oqus 6+, and the red dotted lines represent the Euler angles calculated by E2QKF (without joint angle constraint). Figure 11b and Figure 12b show the Euler angle errors of E2QKF (without joint angle constraint).

As can be seen from Figure 11a and Figure 12a, the Euler angles of E2QKF (without joint angle constraint) overlapped with the Oqus 6+ very closely, which verified the high accuracy of E2QKF (without joint angle constraint). Figure 11b and Figure 12b show that the Euler angle errors of different speeds are maintained within about 5° which verified that E2QKF (without joint angle constraint) can adapt to different motion speeds. Furthermore, during the periods of 10 to 60 s and 60 to 110 s, since the object is doing the repetitive motion, the error in the two periods is also close to repetition, and the overall error remains within a certain angle, indicating that the filter had the potential for long-term human body motion measurement.

#### 3.2.2. Comparing E2QKF (without Joint Angle Constraint) and the Other Two Methods

Yun’s method is an Extended Kalman Filter (EKF) which is integrated with the QUEST algorithm, whilst Zhang’s method is a complementary filter (CF) which is integrated with the ESOQ-2 algorithm, and both of them are able to successfully track the human arm motion in real time under all conditions. To further evaluate the performance of the proposed E2QKF (without joint angle constraint), we selected the two methods for comparison, and we listed each of the Euler angles’ maximum RMSE and the maximum errors of the three methods (three times).

As shown in Table 2, it can be seen that the roll angle’s RMSE of the E2QKF (without joint angle constraint) attitude angle is slightly larger than the CF proposed by Zhang, and the corresponding error indicators are smaller than the other two methods. Although the CF has a simple structure and a small amount of calculation, the accuracy of the E2QKF (without joint angle constraint) algorithm proposed in this paper is higher, and the measurement error is reduced by 14.8% in comparison. The EKF uses the QUEST algorithm to preprocess the data of the accelerometer and magnetometer, which is similar to the design idea of the E2QKF (without joint angle constraint) algorithm proposed in this paper, but the difference is that Yun had not considered the linear acceleration of the human body, so the accuracy of the algorithm is relatively poor. In summary, the E2QKF (without joint angle constraint) algorithm proposed in this paper has a simple structure, and it can not only adapt to different motion speeds of the human body but also has the potential for long-term high-precision human body motion tracking. It is of great value as a human body motion tracking algorithm.

#### 3.2.3. The Effect of the Joint Angle Constraint Method

When the limb movement speed is faster and the test time is longer, the performance test of the algorithm is relatively stricter. Therefore, we selected the test conditions of the limb at the speed of 2 movements per second and the movement time of 100 s to test the effect of the joint angle constraint method.

**Figure 13:**
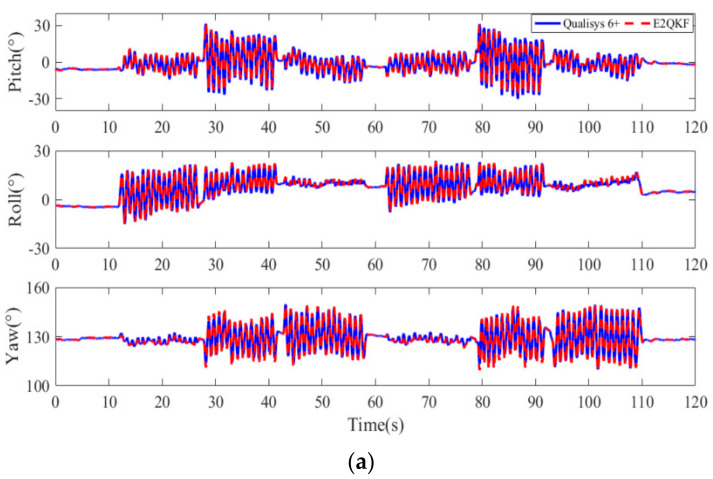

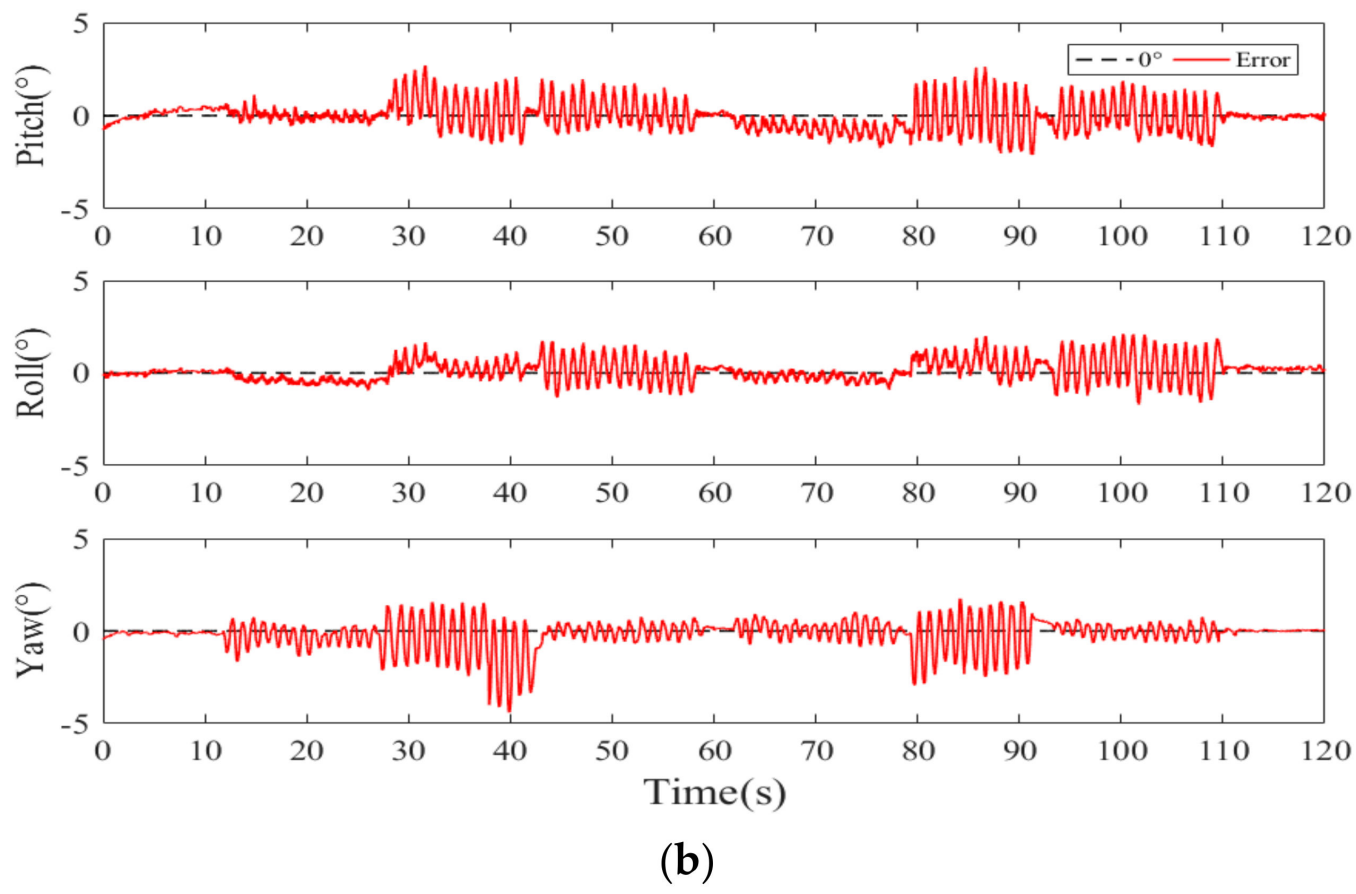
Typical E2QKF (without joint angle constraint) measurement results of the forearm at high speeds. (**a**) The Euler angles of the Oqus 6+ and E2QKF. (**b**) The Euler angle errors.

**Figure 14:**
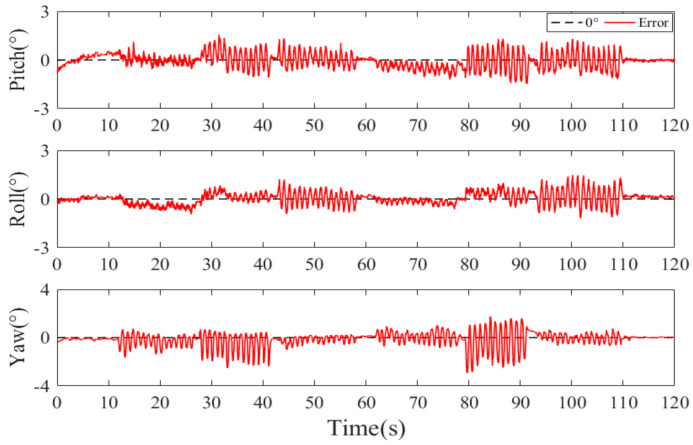
Typical E2QKF (with joint angle constraint) measurement results of the forearm.

Figure 13 and Figure 14 are the typical E2QKF (without joint angle constraint) measurement results of the forearm at 2 movements per second (high speeds). In Figure 13a, the blue solid line represents the motion attitude angle which is measured by the Qualisys 6+ and the red dotted line represents the motion attitude angle calculated by E2QKF (without joint angle constraint). In Figure 13b, the solid red line represents the attitude angle error of E2QKF (without joint angle constraint) relative to the Qualisys 6+. Figure 14 shows the attitude angle error of the forearm of E2QKF (with joint angle constraint) relative to the Qualisys 6+.

As can be seen from Figure 13, the results of the calculation of the motion posture of the forearm are consistent with the accuracy of E2QKF (without joint angle constraint) described above, which further verifies the performance of E2QKF (without joint angle constraint). As can be seen from Figure 14, the error of the attitude calculation of the forearm after the joint angle constraint is reduced compared to before.

Table 3 shows the maximum measurement angle errors of the forearm which were calculated by E2QKF (with/without joint angle constraint). From Table 3, the accuracy of E2QKF combined with joint angle constraints has been further improved: the measurement error for the forearm is reduced by 33.8% when compared to E2QKF (without joint angle constraint). 

## 4. Conclusions

In this paper, a novel Kalman filter was proposed for high-precision human body motion measurement by fusing data from inertial/magnetic sensors. The Kalman filter was designed with the goal of being able to produce accurate orientation estimates of human body motion in real time. Instead of making use of a 7D nonlinear system model, the filter design makes use of a simple first-order linear system model. The Kalman filter was significantly simplified by preprocessing the accelerometer and magnetometer data using the ESOQ-2 algorithm. The compensation of the accelerometer was added in the ESOQ-2 algorithm to improve the accuracy of the ESOQ-2 algorithm in case of rapid human body motion. The quaternion produced by the ESOQ-2 algorithm is provided as the observation vector for the Kalman filter, which reduces the computational complexity. By carrying out the upper arm motion experiments, the performance of E2QKF was verified, the RMSE of the proposed algorithm was less than 2.0° and the maximum error was less than 4.6°, which was better than the advanced Yun’s method and Zhang’s method. Besides, we considered the joint angle constraint, which makes the results more accurate and the maximum error was less than 3.0°. In summary, this paper introduced the proposed method E2QKF for human body motion tracking, and the experimental results illustrated that it is able to track human body motion in real time under different conditions.

## Figures and Tables

**Table 1:** Range of movement angle of upper joints. [24].

Joints	Adduction (°) Abduction (°)	Flexion (°) Extension (°)	Internal Rotation (°) External Rotation (°)
shoulder joint	50**°**	170**°**	45**°**
	90**°**	50**°**	80**°**
elbow joint	0**°**	130**°**	85**°**
	0**°**	0**°**	85**°**
wrist joint	35**°**	90**°**	0**°**
	15**°**	80**°**	0**°**

**Table 2:** Summary of errors.

Euler Angles (°)	E2QKF	CF	EKF
pitch (RMSE)	1.0953	1.8024	1.9024
roll (RMSE)	1.4176	1.0884	1.4793
yaw (RMSE)	1.9574	2.1605	2.5881
Max error	4.596	5.376	9.463

**Table 3:** Comparison of algorithm errors before and after combining joint constraints.

Max Error (°)	E2QKF (Before)	E2QKF (After)
pitch	2.695	1.633
roll	2.117	1.482
yaw	4.402	2.915
